# The Relationship of Latent Toxoplasmosis and Cigarette Smoking: Seroprevalence, Risk Factor, and Case-Control Study in Fars Province, Southern Iran

**DOI:** 10.3390/pathogens11111274

**Published:** 2022-10-31

**Authors:** Mohammad Saleh Bahreini, Sareh Sami Jahromi, Amir Hossein Radfar, Amir Masoud Salemi, Naghmeh Dastan, Qasem Asgari

**Affiliations:** 1 Department of Parasitology and Mycology, School of Medicine, Shiraz University of Medical Sciences, Shiraz 7134845794, Iran; 2 Department of Pathology, School of Medicine, Shiraz University of Medical Sciences, Shiraz 7134845794, Iran

**Keywords:** *Toxoplasma gondii*, cigarette, smoking, dopamine, neurotransmitters

## Abstract

Toxoplasmosis is a parasitic disease with worldwide prevalence. Despite the relatively similar effects of toxoplasmosis and smoking on alteration in neurotransmitters, especially dopamine, little is known about the relation of *Toxoplasma gondii* infection and addiction to cigarette smoking. Therefore, the main objective of this study was to assess the relationship between latent toxoplasmosis and smoking. Through a case-control study, 216 regular cigarette smokers and 324 nonsmoker age- and gender-matched subjects were evaluated for anti-*T.gondii* IgG antibodies with enzyme-linked immunosorbent assay (ELISA). During the sampling, a structured questionnaire was used to obtain the demographic information of participants and the risk factors of acquired *Toxoplasma*. The median ages of case and control groups were 51.04 ± 18.1 (22–97 years) and 51.03 ± 16.5 (21–89 years), respectively (*p* = 0.99). Anti-*T.gondii* IgG antibodies were detected in 44 (20.37%) cases and in 135 (41.67%) controls. There was a statistically significant difference for the positivity rate between the smokers and the control group (OR = 0.35; 95%CI: 0.19–0.65; and *p* = 0.001). The overall prevalence was 33.14%. This study indicated the inverse association between seropositivity to *Toxoplasma* infection and cigarette smoking. This relationship could be due to the changes that latent toxoplasmosis has on the neurotransmitters, especially dopamine, which needs more research.

## 1. Introduction

Toxoplasmosis is a parasitic disease caused by a protozoan called *Toxoplasma gondii*, which belongs to Apicomplexa phylum [1]. It is one of the zoonotic diseases and the infection is spread worldwide. The life cycle of the parasite is completed in two hosts—members of the cat family (Felidae), the only known definitive hosts in which the parasite may undergo sexual reproduction, and human beings and a range of warm-blooded animals known as intermediate hosts [2]. The oral ingestion of tissue cysts containing bradyzoites is a common way for humans to become infected with it. However, it can also be acquired by the ingestion of oocysts that are the products of a sexual cycle in cats [3]. Generally, almost 25 to 30% of humans all over the world are supposed to be infected with *Toxoplasma* [4]. Indeed, the prevalence of the disease is reported to be very diverse between countries (from 10 to 80%). The prevalence is also different in a given country or between different communities in the same region [5]. Due to acute infection or reactivation of chronic infection in immunocompromised individuals and also in congenital transmission, it can cause severe illnesses, such as toxoplasmic encephalitis, and also lead to neuropsychiatric and ophthalmological symptoms. Nevertheless, infection in healthy adults usually causes relatively mild and asymptomatic disease and then alters to the chronic phase form when encystation occurs in organs, especially the brain and muscles [6]. Although chronic toxoplasmosis is asymptomatic in most people, recent studies have suggested that infections with *T. gondii* might have unrecognized effects in hosts [7]. Evidence exists that the parasite is involved in the onset of some behavioral disorders, such as schizophrenia [8,9], bipolar disorder [10], suicidal behavior [11], anxiety disorder [12], and cognitive decline in the elderly [13]. *T. gondii* infection raises inducing neurotransmitter alterations during encystation in the brain [14]. This parasite can dramatically increase the dopamine neurotransmitter by involving the dopaminergic neurons as dopamine-producing nerve cells [15,16,17]. Also, the parasite causes modification in the levels of glutamate, adrenaline, noradrenaline, and serotonin neurotransmitters [18,19]. Numerous aspects of human behavior, such as movement, pleasure, attention, mood, memory, and addiction, are associated with dopamine level in the brain [20,21]. The alteration of dopamine caused by *T. gondii* infection may influence human behaviors [22].

Genetic and behavioral impacts affect the propensity towards substance use [23], but the mechanism of addiction in most cases of substance abuse is associated with increased dopamine transmitter levels in the brain [24]. Studies of the effect of smoking on neurotransmission show that nicotine in cigarettes increases dopamine and norepinephrine and, thus, causes addiction and dependence on cigarette smoking [25]. Moreover, the study by Quattrocki et al. showed that nicotine induces the release of neurotransmitters— dopamine, serotonin, norepinephrine, acetylcholine, and glutamate [26].

While only some studies have evaluated the correlation between *Toxoplasma* infection and substance use, the research represented a positive relationship between latent toxoplasmosis and heroin addiction; the study indicated a possible association between latent toxoplasmosis and the use of opioids [27,28,29]. 

Cigarettes and addictive drugs have an increasing effect on neurotransmitters, such as dopamine in the brain [30]. As *T. gondii* infection potentially affects the same neurotransmitters [31], latent toxoplasmosis might influence the tendency to substance use. According to this hypothesis, this study aimed to investigate the association between *T. gondii* infection and cigarette addiction in a case-control study.

## 2. Materials and Methods

### 2.1. Ethics Approval

The study was confirmed by the ethical review committee of the Shiraz University of Medical Sciences (ethical code: IR.SUMS.MED.REC.1398.006), and informed consent was acquired from the participants. 

### 2.2. Sampling

In this case-control study, all participants were adult males in Shiraz, Fars, Iran, including 216 smokers and 324 nonsmokers. They were chosen and matched in terms of age. The enrollment criteria for the case group were: having the habit of smoking for at least three years at the time of enrollment, and no history of smoking during a lifetime for the control group. Also, individuals who were addicted to other drugs or were drinking alcohol were excluded. Fresh venous blood samples (about 5 mL) were taken from each person and the sera were separated. During the sampling, a structured questionnaire was used to obtain the demographic information of participants and the risk factors of acquired *Toxoplasma*. The questionnaire was completed with information about the participants’ sociodemographic features and potential risk factors for *Toxoplasma*, including having contact with cats. The information obtained from the smokers’ group also included age at the onset of smoking and duration of the addiction. Samples were transferred using the cold chain method of transportation to the laboratory at the Faculty of Medicine at the Shiraz University of Medical Sciences and stored at −20 °C until tested.

### 2.3. ELISA for Detection of IgG Antibodies to Toxoplasma Gondii

The sera samples were tested by the ELISA method through the TOXO IgG kit (ACON ELISA Kit, China), according to the manufacturer’s instructions [32]. Briefly, 100 µL of sera samples was added to each well and the plate was incubated for 30 min at 37 °C. After washing five times with washing buffer, the plate was incubated with the conjugate solution for 30 min at 37 °C (excluding the blank well). After washing, two substrate solutions (50 µL of substrate A and 50 µL of substrate B) were used to visualize the reaction (the color change to blue indicates a positive sample). Then 50 µL stop solution was added to each well. The plate was read at 450–630 nm, using a microplate reader Elx800 (BIO-TEK Instruments, Inc, Winooski, VT, USA). A blank well, calibrator solutions (C1, C2, C3, and C4), and positive and negative control sera were used in each test run. The index value of each specimen was measured by dividing the absorbance value by the calibrator value, based on the manufacturer’s guide. Index value ≤ 0.90 IU/mL was interpreted as negative, ≥1.10 as positive, and 0.91–1.09 as equivocal. The positive and negative controls that were used had been collected from the sera samples in previous studies. The ELISA system used in this study detects anti-*T. gondii* IgG antibody.

### 2.4. Statistical Analysis

Data were analyzed by SPSS software for Windows (version 16, Chicago, IL, USA). Frequency distributions of the independent variables were compared within each group and between them, using the Chi-square test. Simple and multiple logistic regressions were used to assess the relationship between toxoplasmosis and smoking. All variables (i.e., age, place of residence, having domestic house cats as pets, age at onset of smoking, duration of addiction, and seropositivity or seronegativity to toxoplasmosis) were entered into the multiple logistic regression model. *p* < 0.05 was considered as statistically significant. The adjusted odds ratio (OR) and 95% confidence interval were also assessed.

## 3. Results

In this study, the median ages of the case and the control groups were 51.04 ± 18.1 (22–97 years) and 51.03 ± 16.5 (21–89 years), respectively (*p* = 0.99). There were no statistically significant differences among the cases and the controls with respect to age and place of residence. Most participants were greater than 60 years of age in each group. Only 6.9% of persons were residents of rural areas. In this investigation, 44 (20.37%) individuals in the smokers’ group and 135 (41.67%) individuals in the control group were positive for anti-*Toxoplasma* IgG antibody (Table 1). There was a statistically significant difference in the rates of positivity between the smokers’ group and the control group (OR = 0.35; 95% CI: 0.19–0.65; and *p* = 0.001). The difference between seropositivity for *Toxoplasma* and the age group was statistically significant (*p* < 0.05); in smokers, individuals aged 46–60 years showed the highest rate of chronic toxoplasmosis, while none of the smokers under 45 years of age were positive for *Toxoplasma* IgG. In the control group; the >60 age group had the highest positivity rate of anti-*Toxoplasma* IgG antibody. No statically significant difference was observed between test results and keeping a cat using the Chi-square test in groups. There was no equivocal sample in this study. Also, there was no statistically significant association between an IgG positive titer and smoking. Table 2 summarizes the seroprevalence of *Toxoplasma* infection due to sociodemographic characteristics along with logistic regression analysis.

## 4. Discussion

The present research assessed the association between *Toxoplasma gondii* infection and regular cigarette smoking. From the 540 male-only subjects’ screenings (216 cases and 324 controls), the results showed the inverse relationship between regular use of cigarettes and *Toxoplasma gondii* seropositivity in cigarette smokers; seropositivity for anti-*Toxoplasma gondii* IgG was significantly lower than in non-smoker participants. 

Smoking cigarettes regularly increases dependency on them by altering the level of neurotransmitters, especially by raising dopamine in the brain [33]. Also, when *T. gondii* encysts in general areas of the brain, it causes more dopamine release by several mechanisms; two genes in the genome of *Toxoplasma* were found that encode enzymes related to dopamine production in the brain. These enzymes are expressed in the latent phase and bradyzoites form from this [34]. It seems that high dopamine levels in latent toxoplasmosis lead to a decrease in the desire to smoke or the reluctance to continue smoking. Indeed, a smoker with latent toxoplasmosis would keep his or her dopamine levels high without the need for cigarettes. It would reduce the propensity towards smoking, and it would be less likely for non-smokers with latent toxoplasmosis to smoke. This could explain the association between *T. gondii* seropositivity and the reduced desire to use cigarettes. Also, *T. gondii* can more abundantly become encysted in areas of the brain that affect mesolimbic or related regions, all of which are involved in substance use, compared to other brain regions [35,36,37]. Accordingly, the increasing effect of latent toxoplasmosis on dopamine synthesis [15] could decrease substance use in persons with deficient dopaminergic transmission or even in persons with normal dopamine transmission [38,39]. 

Limited studies have investigated the relationship between toxoplasmosis and substance use. However, similar to our study, a study by Andrew et al., in 2018 [22], which examined the prevalence rate of toxoplasmosis in people with self-reported abuse of tobacco, cannabis, methamphetamine, and cocaine among American adults, noted the inverse relationship between the prevalence of *Toxoplasma* infection and self-reported abuse. Also, Teimouri et al., in 2022 [29], reported a negative correlation between *T. gondii* seropositivity and smoking in psychiatric patients. Low seroprevalence of *Toxoplasma* IgG antibody in regular substance users indicated the role of dopamine release through *Toxoplasma* infection in the unwillingness to use.

This result is also justified by the relatively similar mechanisms in elevated dopamine levels of cigarettes and *Toxoplasma.* These mechanisms consist of lowering dopamine receptor levels and reducing striatal monoamine oxidase A (MAO-A) and B (MAO-B) dopamine; dopamine is metabolized in the brain by both MAO-A and MAO-B [40,41].

The current data do not appear to be in compliance with a recent study by Elmorsy et al., 2018 [28], who indicated that *Toxoplasma* infection is significantly related to a high incidence of substance use. This disagreement may be attributed to both studies targeting different communities with differences in number, culture, and residency, differences in the age and sex of the participants, and differences in the classes of the commonly abused drugs. 

Recent studies show that psychiatric disorders, such as schizophrenia, bipolar disorder, and anxiety, are directly related to smoking. Smoking causally increases the risk of developing a number of psychiatric disorders [42,43,44,45]. Also, increasing studies show that latent toxoplasmosis can also be a risk factor in psychiatric diseases [10,45]. The comparison of these findings together shows the parallel effects of smoking and *Toxoplasma* on neurotransmitters [17,46]. Maybe these effects make a person infected with *Toxoplasma* experience little desire to smoke.

Epidemiological studies have shown that *Toxoplasma* infection rate varies widely. The overall seroprevalence of *Toxoplasma* infection in this study was 33.14%. Several studies in Iran reported a seroprevalence range of latent toxoplasmosis approximately close to our seroprevalence range [47,48,49,50,51,52]. Our results showed less seroprevalence than two population-based studies by Mostafavi et al., in 2011 (41.4%) and 2012 (47.5%) [53,54], while it was higher in comparison with the rate among the nomadic populations of Boyer-Ahmad County in Southwestern Iran, where a seroprevalence of 17.3% was reported [55]. Two studies on blood donors in shiraz, by Shaddel et al. and Sarkari et al., indicated a 23.2% and 12.3% seropositivity rate for anti–*T. gondii* IgG antibody, respectively [56,57].

Gharavi et al., in 2018 [58], carried out a large-scale epidemiological study by multistage serum sampling of 882 adolescents from 16 Iran provinces. They found an overall prevalence of 56.3% for *T. gondii* IgG seropositivity, which is dramatically higher than what we found. The diverse prevalence of the disease in various studies indicated that the prevalence of toxoplasmosis is related to broad risk factors, including eating habits, contact with cats, soil-related occupations, age, and interest in animals [59]. 

We also found associations between age and the prevalence of toxoplasmosis in both groups of study: the rate of seropositivity increased with age. This significant difference could be attributed to the increased probability of exposure in older individuals. This result was consistent with other studies’ reports [60,61,62]. 

As it is already known, place of residence and contact with cats are important risk factors for *Toxoplasma* infection [63]. In the present study, no significant differences were found between demographic variables, such as residence area or contact with cats, and seropositivity for *T. gondii* infection. However, as similar results to our results were shown by some other studies [64], these inconsistent reports may be due to the fact that the majority of individuals in this study live in urban areas and most of them had no history of contact with cats.

## 5. Conclusions

In conclusion, due to the higher seroprevalence of the infection in non-smokers compared to smokers, our findings suggest that *Toxoplasma* infection can reduce the urge to smoke. The study of the prevalence of *Toxoplasma* infection in people who were successful in smoking cessation can be useful in confirming the results of this study.

## Figures and Tables

**Table 1:** Median age and frequency to *Toxoplasma* infection in case and control groups.

Test Result	Median Age			Frequency to *Toxoplasma* Infection (%)		
	Case	Control	Total	Case	Control	Total
Positive	63.27	53.64	56.81	44 (20.37%)	135 (41.67%)	179 (33.14%)
Negative	47.9	49.16	48.44	172 (79.63%)	189 (58.33%)	361 (66.86%)
Total	51.04	51.03	51.03	216 (100%)	324 (100%)	540 (100%)

**Table 2:** Univariate and multivariate logistic regression analysis of *Toxoplasma* seropositivity among the smokers’ group and the control group.

Characteristics	Frequency (No.)		Per Cent (%)		*Toxoplasma* Seroprevalence No. (%)		Univariate Analysis		Multivariate Analysis	
	Case	Control	Case	Control	Case	Control	OR (95% CI)	*p* Value	OR (95% CI)	*p* Value
**Residence area**										
Urban	200	303	92.6	93.5	36 (81.8)	126 (93.3%)	2.056 (0.71–5.92)	0.18	1.9 (0.62–5.8)	0.26
Rural	16	21	7.4	6.5	8 (18.2)	9 (6.7%)	1	-	1	-
**Presence of a cat in the household**										
Yes	16	18	7.4	5.6	4 (9.1)	12 (8.9)	0.57 (0.19–1.73)	0.32	0.59 (0.12–1.3)	0.14
No	200	306	92.6	94.4	40 (90.9)	123 (90.1)	1	-	1	-
**Age (Years)**										
<30	40	36	18.5	11.1	0 (0%)	12 (8.9%)	1	-	1	-
31–45	40	93	18.5	28.7	0 (0%)	36 (26.7%)	0.46 (0.13–1.6)	0.22	0.41 (0.11–1.53)	0.18
46–60	64	87	29.6	26.9	24 (54.5%)	33 (24.4%)	0.23 (0.073–0.76)	0.015	0.16 (0.04–0.63	0.008
>60	72	108	33.3	33.3	20 (45.5)	54 (40%)	0.22 (0.071–0.7)	0.011	0.16 (0.042–0.69)	0.01
**Duration of addiction**										
<5	40	-	18.5	-	0 (0%)	-	-	0.99	1	-
6–10	32	-	14.8	-	0 (0%)	-	0.94 (1.6–5.6)	0.95	0.53 (0.04–7.04)	0.63
11–15	28		13		4 (9.1%)		-	0.99	1	-
16–20	12	-	5.6	-	4 (9.1%)	-	3 (0.31–28.8)	0.34	1.73 (0.11–25.5)	0.68
>20	104	-	48.1	-	36 (81.8%)	-	1	-	1	-
**Age at onset of smoking**										
<30	140	-	64.8	-	20 (45.4%)	-	1	-	1	-
31–40	48	-	22.2	-	16 (36.3%)	-	0.33 (0.11–0.98)	0.046	0.55 (0.12–2.6)	0.45
>40	28	-	13	-	8 (18.3)	-	0.41 (0.1–1.6)	0.2	1 (0.11–8.7)	0.99
**Educational level**										
Below high school	68	111	31.5	34.3	20 (29.4%)	51 (37.8%)	0. 27 (0.085–0.86)	0.028	0.63 (0.17–2.3)	0.48
High school	84	117	38.9	36.1	16 (19%)	45 (33.3%)	0.42 (0.13–1.34)	0.144	0.7 (0.2–2.4)	0.57
Bachelor graduates	32	60	14.8	18.5	8 (12.5)	27 (20%)	0.295 (0.084–1.03)	0.057	0.24 (0.06–0.91)	0.03
Higher bachelor’s degree	32	36	14.8	11.1	0 (0%)	12 (8.9%)	1	-	1	-

## Data Availability

The authors confirmed that all the data for this manuscript are available; if someone wants to request the data, they can contact the corresponding author.
